# Prognostic nomograms for gastric carcinoma after surgery to assist decision-making for postoperative treatment with chemotherapy cycles <9 or chemotherapy cycles ≥9

**DOI:** 10.3389/fsurg.2022.916483

**Published:** 2022-08-26

**Authors:** Yifan Li, Xiaojuan Zhang

**Affiliations:** ^1^ Second Department of General Surgery, Chinese Academy of Medical Sciences, Shanxi Province Cancer Hospital, Shanxi Hospital Affiliated to Cancer Hospital, Cancer Hospital Affiliated to Shanxi Medical University, Taiyuan, China; ^2^ Radiology Department, Chinese Academy of Medical Sciences, Shanxi Province Cancer Hospital, Shanxi Hospital Affiliated to Cancer Hospital, Cancer Hospital Affiliated to Shanxi Medical University, Taiyuan, China

**Keywords:** gastric carcinoma, chemotherapy cycles, overall survival, nomogram, risk stratification

## Abstract

**Objective:**

We sought to develop novel nomograms to accurately predict overall survival (OS) of chemotherapy cycles <9 and chemotherapy cycles ≥9 and construct risk stratification to differentiate low-risk and high-risk of two cohorts.

**Methods:**

Patients who underwent curative-intent resection for gastric cancer between January 2002 and May 2020 at a single China institution were identified. Variables associated with OS were recorded and analyzed according to multivariable Cox models. Nomograms predicting 3- and 5-year OS were built according to variables resulting from multivariable Cox models. Discrimination ability was calculated using the Harrell's Concordance Index. The constructed nomogram was subjected to 1,000 resamples bootstrap for internal validation. Calibration curves for the new nomograms were used to test the consistency between the predicted and actual 3- and 5-year OS. Decision curve analysis (DCA) was performed to assess the clinical net benefit. The Concordance index (C-index) and time-dependent receiver operating characteristic (t-ROC) curves were used to evaluate and compare the discriminative abilities of the new nomograms. Finally, prognostic risk stratification of gastric cancer was conducted with X-tile software and nomograms converted into a risk-stratified prognosis model.

**Results:**

For the nomogram predict OS of chemotherapy cycles <9, C-index was 0.711 (95% CI, 0.663–0.760) in internal validation and 0.722 (95% CI, 0.662–0.783) in external validation, which was better than AJCC 8th edition TNM staging (internal validation: 0.627, 95% CI, 0.585–0.670) and (external validation: 0.595,95% CI, 0.543–0.648). The C-index of the nomogram for chemotherapy cycles ≥9 in internal validation was 0.755 (95% CI, 0.728–0.782) and 0.785 (95% CI, 0.747–0.823) in external validation, which was superior to the AJCC 8th edition TNM staging (internal validation: 0.712 95% CI, 0.688–0.737) and (external validation 0.734, 95% CI, 0.699–0.770).The calibration curves, t-ROC curves and DCA of the two nomogram models show that the recognition performance of the two nomogram models was outstanding. The statistical differences in the prognosis among the two risk stratification groups further showed that our model had an excellent risk stratification performance.

**Conclusion:**

This is first reported risk stratification for chemotherapy cycles of gastric carcinoma. Our proposed nomograms can effectively evaluate postoperative prognosis of patients with different chemotherapy cycles of gastric carcinoma. Chemotherapy cycles ≥9 is therefore recommended for high-risk patients with chemotherapy cycles <9, but not for low-risk patients. Meanwhile, combination with multiple therapies are essential to high-risk patients with chemotherapy cycles ≥9 and unnecessary for low-risk patients.

## Introduction

Gastric carcinoma is the fifth most common malignant tumor in the world and the third most common cause of malignant tumor-related death (1). Though the overall survival (OS) of gastric cancer patients has improved with the development of standardized D2 lymphadenectomy (2) and subsequent adjuvant chemotherapy in recent years (3, 4), the long-term survival rate is still unsatisfactory. We should point out that the chemotherapy cycles referred to in this paper includes preoperative neoadjuvant chemotherapy and postoperative adjuvant chemotherapy. However, the importance of chemotherapy cycles in previous studies in prognosis of gastric cancer was often overlooked. In our retrospective study, the mean of total chemotherapy cycles was 9.84 ± 3.80, the mean of postoperative chemotherapy cycles was 9.13 ± 3.98, the mean of chemotherapy cycles of neoadjuvant chemotherapy before surgery was 2.13 ± 3.78, so all cases were categorized by chemotherapy cycles <9 and chemotherapy cycles ≥9. The proportion of postoperative adjuvant chemotherapy was 97.13% (1659/1708) and the proportion of preoperative adjuvant chemotherapy was 12.47% (213/1708). Meanwhile, we found that the 1-year, 3-year and 5 year survival rate of chemotherapy cycles <9 were 90.1% (255/283), 56.8% (155/273), 32.3% (93/288), respectively. The outcomes of 1-year, 3-year and 5 year survival rate of chemotherapy cycles ≥9 were 91.9% (747/813), 60.4% (449/743), 39.0% (279/716), respectively. Nevertheless, the essentiality of chemotherapy cycles in prognosis of gastric carcinoma should not be discounted. It is generally known that chemotherapy is a double—edged sword for patients with cancer. On one hand, chemotherapy can sweep off cancerous cells. On the other hand, there is no denying that chemotherapy drugs can damage normal cells, side effects may occur. In present study, we aimed to developing new novel nomograms that can accurately predict the outcome of patients with gastric carcinoma of chemotherapy cycles <9 and chemotherapy cycles ≥9. Moreover, risk stratification of chemotherapy cycles <9 and chemotherapy cycles ≥9 were applied to distinguish patient who should accomplish adequate chemotherapy and who should avoid overtreatment and who should combine with other treatments. From respective of precision medicine, these will provide accurate guidance of postoperative treatment of gastric carcinoma. Finally, realizing the purpose of personalized treatment for specific patients, and improving the benefit of individual treatment.

## Methods

### Data collection

For the study, researchers analyzed the records of 1,701 individuals who underwent radical gastric surgery and adjuvant chemotherapy for the treatment of gastric cancer at Shanxi cancer hospital from May 2002 to December 2020. A total of 457 gastric cancer patients with less than 9 chemotherapy cycles were selected and randomly divided into groups according to the ratio of 7:3, including 320 cases in the training cohort and 137 cases in the validation cohort. Likewise, a total of 1,244 gastric cancer patients with chemotherapy cycles ≥9 were selected and randomly divided into groups according to the ratio of 7:3, including 858 cases in the training cohort and 385 cases in the validation cohort.

**Figure 1:**
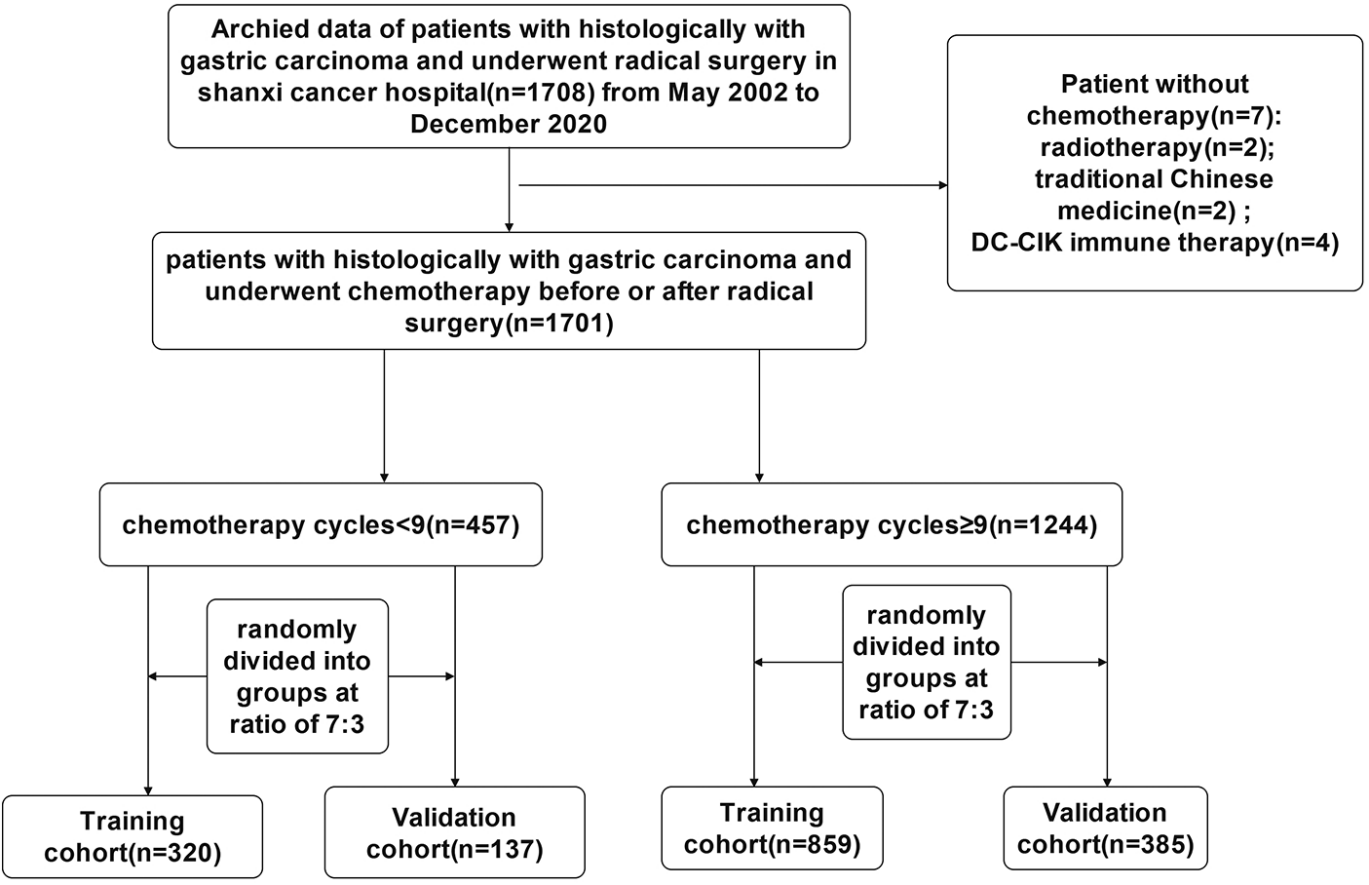
The flowchart of study population enrolment in the training and validation cohort of gastric cancer.

Inclusion criteria: (1) gastric cancer confirmed by histological pathology; (2) adjuvant chemotherapy after curative gastrectomy; (3) complete clinicopathological and follow-up data (all biomarkers were measured within 1 week before surgery); (4) no severe organ damage after surgery; (5) no other malignant tumors, no cause of death other than GC. Exclusion criteria: (1) merging other systemic tumors; (2) lacking or incomplete clinical data; (3) Palliative surgery or bypass surgery; (4) Pathological classification confirmed as non-gastric cancer. Tumor stage was reclassified according to the AJCC 8th TNM classification. The informed consent statements of patients could not be obtained because the study was a retrospective analysis. The procedure for this study was reviewed and approved by the Ethics Committee of Shanxi cancer hospital. The study complied with the standards of the Declaration of Helsinki, and patient data were anonymous and strictly confidential. A flowchart of the detailed research process is shown in Figure 1.

To be included in the study, the patients had to meet a set of requirements that included the primary histologically confirmed diagnosis of gastric cancer and curative-intent surgery (R0–R1). The factors that affected the outcome of the surgery were identified. Some of these included gender, age at surgery, vascular invasion, neural invasion, pT stage, number of positive lymph nodes, TNM stage (according to the 8th edition of the American Joint Committee), Lauren classification, maximum tumor diameter, type of gastrectomy, omentum metastasis, surgical margin, chemotherapy administration, chemotherapy regimen, multiple organ resection, histological classification, Clavien-Dindo classification for complication, expression of AE1/AE3, Ki67, CK7, CK20, CDX-2, SATB-2, SYN, CGA, CD56, MLH1, PMS2, Her-2, MSH2 and MSH6, overall survival (OS). The number of chemotherapy cycles, including the number of neoadjuvant chemotherapy and postoperative chemotherapy data, surgical records, medical records and follow-up data were retrospectively analyzed. It is noteworthy that chemotherapy cycles, including the neoadjuvant chemotherapy cycles and postoperative chemotherapy cycles. The follow-up time was calculated using the electronic records of the patients' visits to the hospital and the oncologist. The follow-up time was calculated based on the date when the patients last visited the hospital and the date when they last contact with the surgeon. Overall survival (OS) was calculated based on the time from the surgery to death or the last follow-up. This research study was conducted retrospectively from data obtained for clinical purposes. All treatments were performed in accordance with institutional guidelines and regulations. Our study obtained informed consent from the study participants, in accordance with the Declaration of Helsinki.

### Statistical analysis

Descriptive statistics are expressed as absolute numerical percentum for categorical variables and continuous variables' medians were derived as medians of the interquartile range. Kaplan-Meier curves were used to present OS. Factors related to OS were analyzed and noted in accordance with multivariate Cox regression. The results were presented as hazard ratios, 95% CI, and *P* values. *P* < 0.05 was considered statistically significant. Data processed using various software, including R software (version 4.1.2), SPSS 25.0, and Grand pad Prism 9.3.

### Nomogram performance

The primary goal of this study was to create nomograms to predict OS. The parameters used for the model were derived from the Cox model. The nomograms were computed by the Cox regression of various parameters associated with the OS. The performance of the models was then computed through 10,000 repetitions. The performance of the nomogram was evaluated through the calibration and discrimination tests. The discrimination test was carried out by Harrell's concordance index (C-Index), which was the agreement between the predictions and observations. The calibration curves were performed to evaluate the nomogram's discrimination. The area under the receiver operating characteristic curve was then used to test the model's discrimination. The value of AUC was 0.5–0.7 suggested that inferior discrimination of the model; 0.7–0.9 suggested that middling performance of the model; and >0.9 indicated excellent performance. The calibration curves were used to test the consistency of the results. Decision curve analysis (DCA) was then performed to assess the clinical benefit (5–7).

## Results

### Basic characteristics of training cohort of chemotherapy cycles <9 and chemotherapy cycles ≥9

In the training cohort, we included 320 gastric cancer patients with chemotherapy cycles <9, of whom 134 (41.9%) died. In training cohort of chemotherapy cycles ≥9 including 859 gastric cancer patients, of whom 310 (36.1%) died (Table 1).

**Table 1:** Baseline clinical features.

Variables	Chemotherapy cycle <9: Training cohort (*n* = 320)	Chemotherapy cycle ≥9: Training cohort (*n* = 859)
	Mean ± SD/No (%)	Mean ± SD/No (%)
Gender		
Male	249 (77.8%)	703 (81.8%)
Female	71 (22.2%)	156 (18.2%)
Age (year)	56.00 ± 10.11	59.67 ± 9.67
pT stage		
T1	29 (9.1%)	193 (22.5%)
T2	17 (5.3%)	38 (4.4%)
T3	116 (35.3%)	226 (26.3%)
T4	161 (50.3%)	402 (46.8%)
Number of positive lymph nodes		
0	74 (23.1%)	325 (37.8%)
1–2	75 (23.4%)	156 (18.2%)
3–6	54 (16.9%)	115 (13.4%)
≥7	117 (36.6%)	263 (30.6%)
TNM Stage		
I	31 (9.7%)	210 (24.4%)
II	94 (29.4%)	194 (22.6%)
III	181 (56.6%)	428 (49.8%)
V	14 (4.4%)	27 (3.1%)
Vascular invasion		
Negative	143 (44.7%)	419 (48.8%)
Positive	177 (55.3%)	440 (51.2%)
Neural invasion		
Negative	145 (45.3%)	479 (55.8%)
Positive	175 (54.7%)	380 (44.2%)
Lauren classification		
Intestinal	107 (33.4%)	362 (42.1%)
Diffuse	125 (39.1%)	290 (33.8%)
Mixed	88 (27.5%)	207 (24.1%)
Maximum diameter of Tumor (cm)		
<6	191 (59.7%)	573 (66.7%)
≥6	129 (40.3%)	286 (33.3%)
Type of gastrectomy		
Primal	35 (10.9%)	90 (10.5%)
Distal	100 (31.3%)	279 (32.5%)
Total	185 (57.8%)	490 (57.0%)
Omentum metastasis		
Negative	310 (96.9%)	838 (97.6%)
Positive	10 (3.1%)	21 (2.4%)
Surgical margin		
Negative	301 (94.1%)	819 (95.3%)
Positive	19 (5.9%)	40 (4.7%)
Her-2		
(−)	196 (61.3%)	534 (62.2%)
(+)	124 (38.7%)	325 (37.8%)
Multiple organ excision		
No	301 (94.1%)	822 (87.2%)
Yes	19 (5.9%)	37 (12.8%)
Clavien-Dindo classification for complication		
Grade I–II	278 (86.9%)	749 (87.2%)
Grade III–V	42 (13.1%)	110 (12.8%)
Histological classification		
Adenocarcinoma	262 (81.9%)	702 (83.8%)
Others	58 (18.1%)	157 (18.2%)
AE1/AE3		
Negative	42 (13.1%)	209 (24.3%)
Positive	278 (86.9%)	650 (75.7%)
Ki67 (%)	61.89 ± 22.72	53.29 ± 27.57
CK7		
Negative	149 (46.6%)	444 (51.7%)
Positive	171 (53.4%)	415 (48.3%)
CK20		
Negative	228 (71.3%)	624 (72.6%)
Positive	92 (28.7%)	235 (27.4%)
CDX-2		
Negative	165 (51.6%)	491 (57.2%)
Positive	155 (48.4%)	368 (42.8%)
SATB-2		
Negative	250 (78.1%)	714 (83.1%)
Positive	70 (21.9%)	145 (16.9%)
SYN		
Negative	225 (70.3%)	641 (74.6%)
Positive	95 (29.7%)	218 (25.4%)
CGA		
Negative	269 (84.1%)	689 (80.2%)
Positive	51 (15.9%)	170 (19.8%)
CD56		
Negative	188 (58.8%)	563 (65.5%)
Positive	132 (41.2%)	296 (34.5%)
MLH1		
Negative	27 (8.4%)	124 (14.4%)
Positive	293 (91.6%)	735 (85.6%)
PMS2		
Negative	65 (20.3%)	272 (31.7%)
Positive	255 (79.7%)	587 (68.3%)
MSH2		
Negative	35 (10.9%)	128 (14.9%)
Positive	285 (89.1%)	731 (85.1%)
MSH6		
Negative	29 (9.1%)	119 (13.9%)
Positive	291 (90.9%)	740 (86.1%)
Overall survival (months)	40.66 ± 21.22	39.10 ± 23.36
Progression-free survival (months)	31.58 ± 22.90	35.75 ± 24.31
Status		
Censored	186 (58.1%)	549 (63.9%)
Mortality	134 (41.9%)	310 (36.1%)

### Development and validation of the prediction model of OS of chemotherapy cycles <9

Multivariate Cox regression analysis from Table 2 showed that age, number of positive lymph nodes, omentum metastasis, multiple organ resection, Clavien-Dindo classification for complication were independent risk factors of overall survival (OS) of training cohort of chemotherapy cycles <9 (*n* = 320).

**Table 2:** Multivariate analysis of OS of training cohort of chemotherapy cycle <9 and analyzed by Cox regression.

Variables	B	SE	Wald	df	*P*	HR	95% CI
Age	0.024	0.010	5.752	1	0.016	1.025	1.004–1.045
Number of positive lymph nodes			9.829	3	0.020		
0 vs. 1–2	−0.091	0.357	0.065	1	0.799	0.913	0.453–1.839
0 vs. 3–6	0.244	0.390	0.392	1	0.531	1.277	0.595–2.741
0 vs. ≥7	0.789	0.357	4.886	1	0.027	2.201	1.094–4.430
Omentum metastasis							
Negative vs. Positive	1.148	0.430	7.140	1	0.008	3.153	1.358–7.319
Multiple organ excision							
No vs. Yes	0.975	0.301	10.494	1	0.001	2.652	1.470–4.783
Clavien-Dindo classification for complication							
Grade I–II vs. Grade III–V	0.631	0.269	5.480	1	0.019	1.879	1.108–3.187

B, regression coefficient; SE, standard error; df, degree of freedom;HR, hazard ratio; CI, confidence interval.

**Figure 2:**
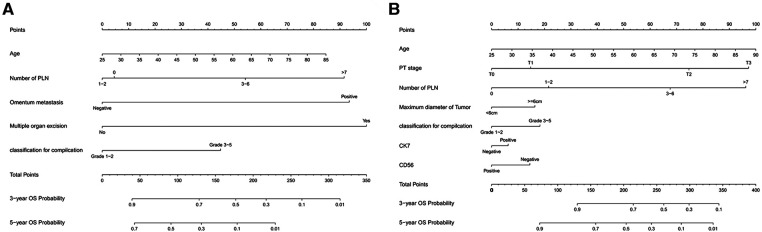
(**A**) Nomogram model to predict 3-year and 5-year OS of chemotherapy cycles <9 and (**B**) Nomogram model to predict 3-year and 5-year OS of chemotherapy cycles ≥9.

Age, number of positive lymph nodes, omentum metastasis, multiple organ resection, Clavien-Dindo classification for complication were incorporated into the nomogram of chemotherapy cycles <9. The nomogram predicted the survival probability of 3-year and 5-year overall survival (OS) in patients of gastric cancer with less than 9 chemotherapy cycles. The nomogram model can be used to predict a favorable outcome for a patient's 3-year and 5-year overall survival (OS). It combines the various factors that are known to predict the likelihood of patients achieving a favorable outcome. Figure 2A exhibited that the nomogram model is capable of predicting a favorable outcome for patients with gastric cancer by combining the various factors affect 3- and 5-year OS of patients. In the training cohort, the C-index for OS chemotherapy cycles <9 was 0.711 (95% CI, 0.663–0.760). The nomogram was compared with the discrimination of AJCC 8th edition TNM staging, the C-index of the nomogram was better than AJCC 8th edition TNM staging (0.627, 95% CI, 0.585–0.670).

### Validation the predictive accuracy of nomograms for OS of chemotherapy cycles <9

**Figure 3:**
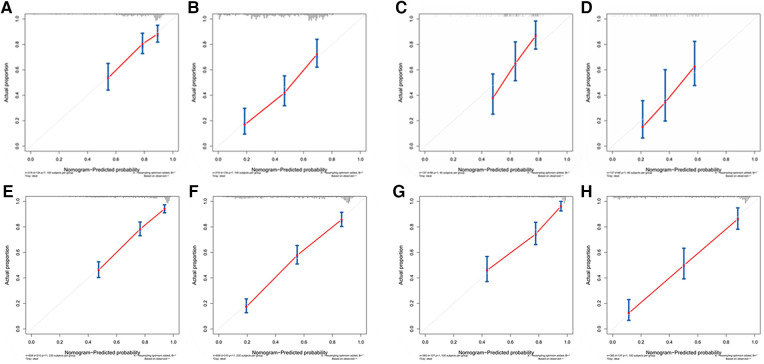
(**A**) Calibration curves of internal validation to predict 3- year OS of chemotherapy cycles <9, (**B**) Calibration curves of external validation to predict 3- year OS of chemotherapy cycles <9, (**C**) Calibration curves of internal validation to predict 5- year OS of chemotherapy cycles <9, (**D**) Calibration curves of external validation to predict 5- year OS of chemotherapy cycles <9, (**E**) Calibration curves of internal validation to predict 3- year OS of chemotherapy cycles ≥9, (**F**) Calibration curves of external validation to predict 3- year OS of chemotherapy cycles ≥9, (**G**) Calibration curves of internal validation to predict 5- year OS of chemotherapy cycles ≥9 and (**H**) Calibration curves of external validation to predict 5- year OS of chemotherapy cycles ≥9.

**Figure 4:**
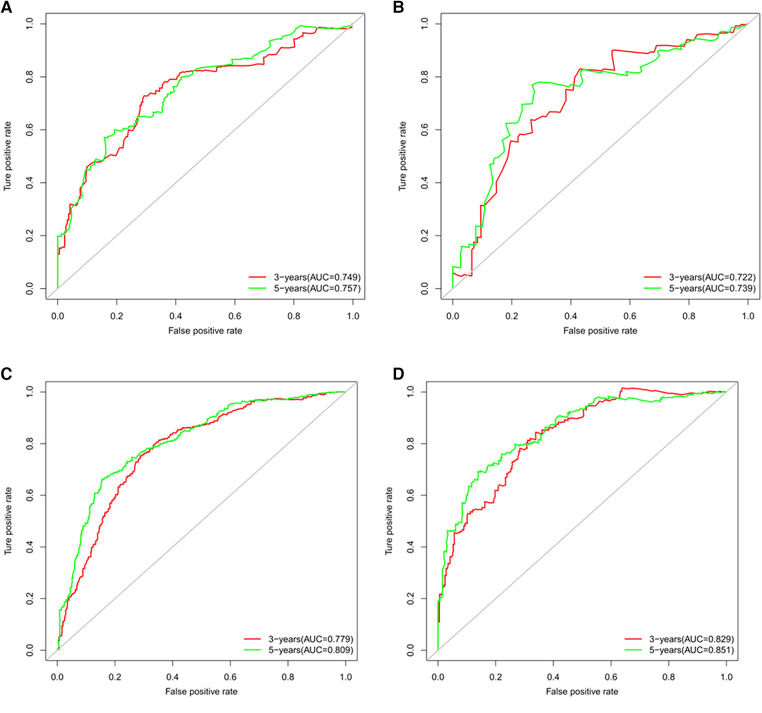
(**A**) Time-dependent receiver operating characteristic (t-ROC) curves of internal validation to predict OS of chemotherapy cycles <9, (**B**) Time-dependent receiver operating characteristic (t-ROC) curves of external validation to predict OS of chemotherapy cycles <9, (**C**) Time-dependent receiver operating characteristic (t-ROC) curves of internal validation to predict OS of chemotherapy cycles ≥9 and (**D**) Time-dependent receiver operating characteristic (t-ROC) curves of external validation to predict OS of chemotherapy cycles ≥9.

**Figure 5:**
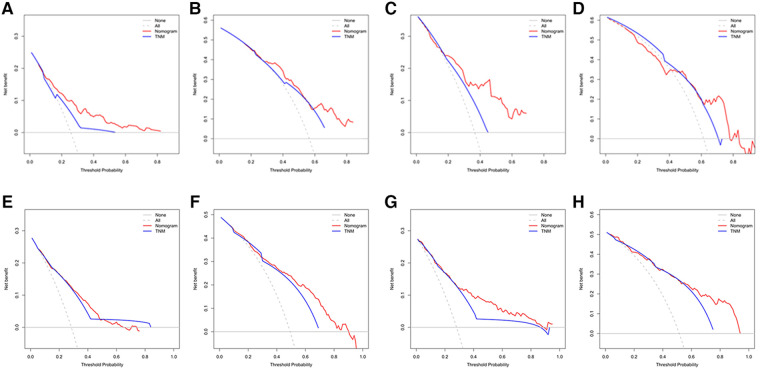
(**A**) Decision curve analysis (DCA) of internal validation to predict 3- year OS of chemotherapy cycles <9, (**B**) Decision curve analysis (DCA) of internal validation to predict 5- year OS of chemotherapy cycles <9, (**C**) Decision curve analysis (DCA) of external validation to predict 3- year OS of chemotherapy cycles <9, (**D**) Decision curve analysis (DCA) of external validation to predict 5- year OS of chemotherapy cycles <9, (**E**) Decision curve analysis (DCA) of internal validation to predict 3- year OS of chemotherapy cycles ≥9, (**F**) Decision curve analysis (DCA) of internal validation to predict 5- year OS of chemotherapy cycles ≥9, (**G**) Decision curve analysis (DCA) of external validation to predict 3- year OS of chemotherapy cycles ≥9 and (**H**) Decision curve analysis (DCA) of external validation to predict 5- year OS of chemotherapy cycles ≥9.

Figures 3A–D showed that the calibration curves for the predicted probability of OS of 3, 5 years from internal validation and external validation of the nomogram predictions are in line with actual observations. Time-dependent ROC in the internal validation showed favorable discriminations and the area under the curve (AUC) of 3-year OS and 5-year OS were 0.749 (95% CI, 0.665–0.814), 0.722 (95% CI, 0.696–0.880), respectively. In addition, AUC of 3-year OS and 5-year OS in the external validation were 0.757 (95% CI, 0.658–0.821), 0.739 (95% CI, 0.646–0.892), respectively (Figures 4A,B). To evaluate the potential clinical benefit of our nomogram model, we performed a decision analysis curve (DCA) to compare the difference in 5- and 3-year OS between the AJCC 8th edition TNM staging and the nomogram. The model's internal validation C-index was 0.711 (95% CI, 0.663–0.760), which was superior to the AJCC 8th edition TNM staging C-index (0.627, 95% CI, 0.585–0.670). The external validation C-index was 0.722 (95% CI, 0.662–0.783), and excelled the AJCC 8th edition TNM staging (0.595,95% CI, 0.543–0.648). Both C-index of external validation and external validation were higher than AJCC 8th edition TNM stage, indicating that the model showed good predictive ability (Figures 5A–D).

### Risk scoring of stratification system of OS for chemotherapy cycles <9

**Figure 6:**
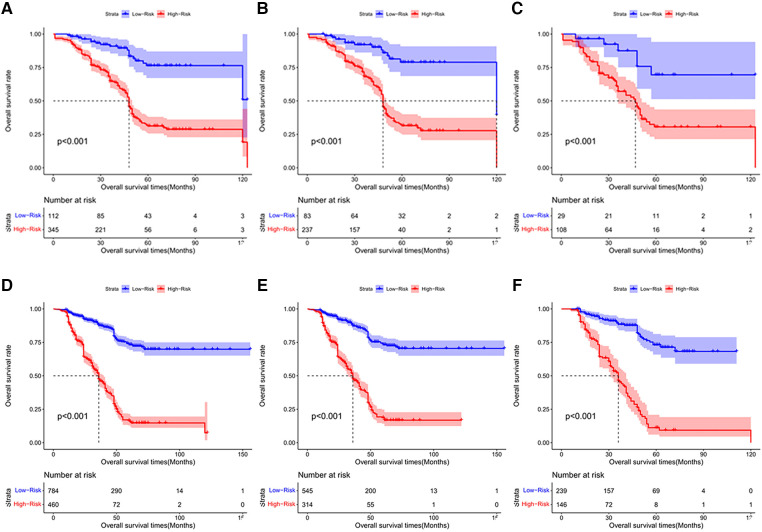
The Kaplan-Meier survival curves for patients with different scores who underwent chemotherapy cycles <9. (**A**) all cohort, (**B**) training cohort and (**C**) validation cohort; The Kaplan-Meier survival curves for patients with different scores who underwent chemotherapy cycles ≥9. (**D**) all cohort E. training cohort F. validation cohort.

According to the final nomogram model of chemotherapy cycles <9, each patient is calculated and scored. We based on the cut-off value of overall survival (OS) scores for the training cohort (*n* = 320) generated by the X-tile software. The log-rank test method was used to compare survival times among the different risk groups. Total scores were calculated according to the prognostic nomogram. Based on cutoff value of 49.72, the overall cohort (*n* = 457) was divided into 2 groups with completely different survival risk probabilities (Figure 6): the low-risk group [0 ≤ 49.72, including 83 patients in the training sequence (*n* = 320) and 29 patients in the validation sequence (*n* = 137)], and the high-risk group [>49.72, including 237 patients in the training sequence (*n* = 320) and 108 patients in the validation sequence (*n* = 137)]. Figure 6 shows OS curves stratified by risk scores for all cohorts, training cohorts, and validation cohorts, with P values less than 0.001 for all three cohorts. The median OS of low-risk group in all cohort (*n* = 457) was not reached and the median OS was 48 months in the high-risk group. The median OS in the low-risk group and the high-risk group in the training cohort (*n* = 320) was 120 months and 48 months, respectively. The median OS of low-risk group for validation cohort (*n* = 137) had not yet reached and the median OS was 47 months in the high-risk group. Statistical differences in prognosis between the two risk stratification groups further suggested that our model has good risk stratification performance.

### Development and validation of the prediction model of OS of chemotherapy cycles ≥9

Multivariate Cox regression analysis from Table 3 displayed that number of positive lymph nodes, age, pT stage, maximum diameter of tumor, Clavien-Dindo classification for complication, expression of CK7 and CD36 identified independent risk factors of overall survival (OS) for training cohort (*n* = 859) of chemotherapy cycles ≥9.

**Table 3:** Multivariate analysis of OS of training cohort of chemotherapy cycle ≥9 and analyzed by Cox regression.

Variables	B	SE	Wald	df	*P*	HR	95% CI
Age	0.020	0.005	14.518	1	<0.001	1.020	1.010–1.031
pT stage			19.339	3	<0.001		
T1 vs. T2	0.024	0.529	0.002	1	0.963	1.025	0.363–2.890
T1 vs. T3	0.779	0.329	5.618	1	0.018	2.180	1.144–4.152
T1 vs. T4	1.159	0.331	12.287	1	<0.001	3.187	1.667–6.093
Number of positive lymph nodes			24.885	3	<0.001		
0 vs. 1–2	0.307	0.201	2.346	1	0.126	1.360	0.918–2.015
0 vs. 3–6	0.624	0.216	8.356	1	0.004	1.867	1.223–2.850
0 vs. ≥7	0.936	0.210	19.839	1	<0.001	2.549	1.689–3.848
Maximum diameter of Tumor							
<6 cm vs. ≥6 cm	0.274	0.109	6.390	1	0.011	1.316	1.064–1.627
Clavien-Dindo classification for compilation							
Grade I–II vs. Grade III–V	0.423	0.125	11.416	1	0.001	1.527	1.195–1.952
CK7							
Negative vs. Positive	0.244	0.115	4.485	1	0.034	1.276	1.018–1.598
CD56							
Negative vs. Positive	−0.314	0.121	6.691	1	0.010	0.731	0.576–0.927

B, regression coefficient; SE, standard error; df, degree of freedom;HR, hazard ratio; CI, confidence interval.

The nomogram predicted the survival probability of 3-year and 5-year overall survival (OS) in patients of gastric cancer with less than 9 chemotherapy cycles. The model was powered by seven factors: number of positive lymph nodes, age, pT stage, maximum diameter of tumor, Clavien-Dindo classification for complication, expression of CK7 and CD36. A nomogram model is a tool that combines the various factors that can predict the 3-year and 5-year overall survival (OS) of patients with gastric cancer. It can be used to identify those with a better chance of achieving a favorable outcome. Based on Figure 2B, the nomogram model combining with the above independent predictors to predict 3- and 5-year OS. In the training cohort, the C-index predicted of PFS was 0.755 (95% CI, 0.728–0.782). The nomogram was compared with the discrimination of AJCC 8th edition TNM staging, which was precede C-index of the AJCC 8th edition TNM staging (0.712, 95% CI, 0.688–0.737).

According to Figures 3E–H, calibration curves of the internal and external validations showed that the nomogram's predictions are consistent with the actual observations. Then the time-dependent ROC curve was constructed to evaluate the predictive accuracy of the nomogram. The area under the curve (AUC) of the time-dependent receiver operating character (t-ROC) curves is computed for the validation of the model's 3-year and 5-year OS, with the AUC of 0.779 (95% CI, 0.746–0.823) for the internal validation of 3-year OS and the AUC for external validation of 3-year OS was 0.829 (0.779–0.882). For 5-year OS, the AUC of internal verification was 0.809 (95% CI, 0.790–0.869) and the AUC of external verification was 0.851 (95% CI, 0.792–0.907). The values of the AUC are greater than the internal and external verification values, which indicated that the model is outstanding (Figures 4C,D).

The decision analysis curves (DCA) performed on the data revealed the potential clinical benefit of our nomogram (Figures 5E–H). It also showed that our nomogram performed better than the AJCC TNM classification. Regardless of the training or validation cohort, our nomogram had a larger net benefit than the AJCC TNM staging. C-index of the internal validation was 0.755 (95% CI, 0.728–0.782), which was better than C-index of the AJCC 8th edition TNM staging (0.712, 95% CI, 0.688–0.737). In externally validation, C-index was 0.785 (95% CI, 0.747–0.823), which was higher than C-index of the AJCC 8th edition TNM staging (0.734, 95% CI, 0.699–0.770).

### Risk scoring of stratification system of OS for chemotherapy cycles ≥9

Based on the final nomogram model, each patient is calculated their score. Our cutoff value for overall survival (OS) of training cohort (*n* = 859) generated by the X-tile software. The log-rank test method was used to compare survival times among the different risk groups. Total scores were calculated according to the prognostic nomogram. According to the cutoff value of 215.53, the training cohort (*n* = 859) was divided into 2 groups with totally different overall survival (OS) risk probabilities (Figures 6D–F): the low-risk group [0 ≤ 213.53, including 545 patients in the training cohort (*n* = 859) and 239 patients in the validation cohort (*n* = 385)], and the high-risk group [>213.53, including 314 patients in the training cohort (*n* = 859) and 146 patients in the validation cohort (*n* = 385)]. Figure 6 shows overall survival curves stratified by risk scores for all cohorts, training cohorts, and validation cohorts, with P values less than 0.001 for all three cohorts. The median OS of low-risk group in all cohort (*n* = 1244), training cohort (*n* = 859), and validation cohort (*n* = 385) have not reached and the median OS of high-risk group of three cohort were identical, 36 months. Statistical differences in prognosis between the two risk stratification groups further indicated that our model has good risk stratification performance.

## Discussion

Although the number of chemotherapy cycles needed to achieve an oncologic benefit has been acknowledged, little attention paid to chemotherapy cycles in prognosis of gastric cancer. A research in Korean concentrates on the minimum number of cycles that patients should complete to achieve an oncologic advantage in the treatment of gastric cancer. The researchers discovered that the patients who completed fewer than four cycles had a lower disease-free survival rate (8). In my previous research, the relationship between chemotherapy cycles and recurrence of gastric cancer had been discussed. This research demonstrated that ensuring the number of chemotherapy cycles >9 was enable to reduce the rate of recurrence and ameliorate the prognosis of gastric cancer. Likewise, chemotherapy cycles <9 may increase probability of recurrence and caused deterioration in OS (9).

In our study, we incorporated various factors, including clinical characteristics, pathological parameters, and tumor molecular markers, and applied the COX regression to select variables among them. We finally established a reliable prognosis nomogram of chemotherapy cycles <9 to predict 3-year, 5-year overall survival, which contained five indicators: age, the number of positive lymph nodes, omentum metastasis, multiple organ resection, and Clavien-Dindo postoperative complication classification. In the same way, a nomogram for predicting OS of chemotherapy cycles ≥9 was also developed, including seven variables, number of positive lymph nodes, age, pT stage, maximum tumor diameter, Clavien-Dindo postoperative complication grade, expression of CK7 and CD56. Due to some factors that might cause differences in the prognosis of patients after radical gastrectomy in different centers, we decided to conduct both internal and external validations to evaluate the performance of our model more comprehensively. After internal and external validations, the model demonstrated predication performance with satisfying calibration, discrimination, and clinical utility. In addition, we performed a population-based analysis to classify the patients into two risk groups, the nomogram was further improved into a risk-stratified prognosis model. This allowed us to improve the nomogram's performance and create a risk-stratified prognosis model. The goal of this study was to provide guidance to clinicians and improve communication between patients and their doctors.

At present, the most widely used prognostic risk prediction system of clinical application is TNM staging, but its value has visibly deteriorated result from the limitations of its accuracy and stability (10–12). A number of studies have shown the potential of nomograms to improve the quality of care and reduce the number of unnecessary tests for patients with gastric cancer (13). Researchers have explored a considerable number of prognostic factors related to gastric cancer, such as age, sex, tumor size, number of positive lymph nodes, depth of invasion, tumor location, Lauren classification, histologic classification, and biological markers. As a result, various models associated with prognosis established subsequently (14–17).

We explored the two prognostic nomograms models to predict and perform risk stratification to distinguish different risk degree for patients with chemotherapy cycles <9 and chemotherapy cycles ≥9. For instance, a patient with chemotherapy cycles <9, we calculated the score and discriminate the risk stratification of the patient. If the result is low-risk, adequate chemotherapy cycles is dispensable. Likewise, if the result is high-risk, it is crucial to accomplish adequate chemotherapy cycles (≥9). Also, a patient with chemotherapy cycles ≥9, it unnecessary for low-risk patients to accept other postoperative treatment and combination chemotherapy with other remedies is essential, such as radiotherapy, targeted therapy, immunotherapy.

Despite the promising findings, the current study has several limitations that should be noted. First, the training cohort and validation cohort involved in model construction and validation are from a single center, which requires further validation from data from other medical centers; second, the prediction effect of 3-year OS of the two nomogram models was similar in AJCC 8th edition TNM stage and further research is needed; third, this study did not distinguish between early stage and advanced gastric cancer patients, and there may be differences in the prediction performance of chemotherapy cycles <9 and chemotherapy cycles ≥9 or different stages of gastric cancer patients.

We noticed that the studies on the relationship between chemotherapy cycles and prognosis of gastric cancer were rare. What's more, we need more samples from other research institute to ensure the exactitude and accuracy of nomogram to predict and improve prognosis of gastric cancer.

## Conclusion

This is first reported risk stratification for chemotherapy cycles of gastric carcinoma. Our proposed nomograms can effectively evaluate postoperative prognosis of patients with different chemotherapy cycles of gastric carcinoma. Chemotherapy cycles ≥9 is therefore recommended for high-risk patients with chemotherapy cycles <9, but not for low-risk patients. Meanwhile, combination with multiple therapies are essential to high-risk patients with chemotherapy cycles ≥9 and unnecessary for low-risk patients.

## Data Availability

The original contributions presented in the study are included in the article/Supplementary Material, further inquiries can be directed to the corresponding author/s.

## References

[1] Torre LA Bray F Siegel RL Ferlay J Lortet-Tieulent J Jemal A. Global cancer statistics, 2012. CA Cancer J Clin. (2015) 65:87–108. 10.3322/caac.21262 25651787

[2] Songun I Putter H Kranenbarg EM Sasako M van de Velde CJ. Surgical treatment of gastric cancer: 15-year follow-up results of the randomised nationwide Dutch D1D2 trial. Lancet Oncol. (2010) 11:439–49. 10.1016/S1470-2045(10)70070-X 20409751

[3] Sakuramoto S Sasako M Yamaguchi T Kinoshita T Fujii M Nashimoto A Adjuvant chemotherapy for gastric cancer with S-1, an oral fluoropyrimidine. N Engl J Med. (2007) 357:1810–20. 10.1056/NEJMoa072252 17978289

[4] Bang YJ Kim YW Yang HK Chung HC Park YK Lee KH Adjuvant capecitabine and oxaliplatin for gastric cancer after D2 gastrectomy (CLASSIC): a phase 3 open-label,randomised controlled trial. Lancet. (2012) 379:315–21. 10.1016/S0140-6736(11)61873-4 22226517

[5] Spolverato G Capelli G Lorenzoni G Gregori D Squires MH Poultsides GA Development of a prognostic nomogram and nomogram software application tool to predict overall survival and disease-free survival after curative-intent gastrectomy for gastric cancer. Ann Surg Oncol. (2022) 29(2):1220–9. 10.1245/s10434-021-10768-7 34523000

[6] Zhang M Ding C Xu L Feng S Ling Y Guo J A nomogram to predict risk of lymph node metastasis in early gastric cancer. Sci Rep. (2021) 11(1):22873. 10.1038/s41598-021-02305-z 34819570 PMC8613278

[7] Yuan H-L Zhang X Li Y Guan Q Chu WW Yu HP A nomogram for predicting risk of thromboembolism in gastric cancer patients receiving chemotherapy. Front Oncol. (2021 May 26) 11:598116. 10.3389/fonc.2021.598116 34123774 PMC8187914

[8] Jeong S-H Yoo M-W Son Y-G Oh SJ Kim JH Kim HI Appropriate number of adjuvant chemotherapy cycles for patients with stage 2 or 3 gastric cancer after curative gastrectomy: a multicenter cohort study. Ann Surg Oncol. (2021) 28(8):4458–70. 10.1245/s10434-020-09504-4 33423177

[9] Li Y Zhao H. Postoperative recurrence of gastric cancer depends on whether the chemotherapy cycle was more than 9 cycles: based on a retrospective and observational study of follow-up within 3 years of 843 patients. Medicine (Baltimore). (2022) 101(5):e28620. 10.1097/MD.0000000000028620 35119006 PMC8812606

[10] Lu J Zheng ZF Wang W Xie JW Wang JB Lin JX A novel TNM staging system for gastric cancer based on the metro-ticket paradigm: a comparative study with the AJCC-TNM staging system. Gastric Cancer. (2019) 22(4):759–68. 10.1007/s10120-018-00904-w 30612230

[11] Zhou YY Kang YT Chen C Xu FF Wang HN Jin R. Combination of TNM staging and pathway based risk score models in patients with gastric cancer. J Cell Biochem. (2018) 119(4):3608–17. 10.1002/jcb.26563 29231991

[12] Lu J Zheng ZF Xie JW Wang JB Lin JX Chen QY Is the 8th edition of the AJCC TNM staging system sufficiently reasonable for all patients with noncardia gastric cancer? A 12,549-patient international database study. Ann Surg Oncol. (2018) 25(7):2002–11. 10.1245/s10434-018-6447-0 29725896

[13] Iasonos A Schrag D Raj GV Panageas KS. How to build and interpret a nomogram for cancer prognosis. J Clin Oncol. (2008) 26(8):1364–70. 10.1200/JCO.2007.12.9791 18323559

[14] Necchi A Sonpavde G Lo Vullo S Giardiello D Bamias A Crabb SJ Nomogram-based prediction of overall survival in patients with metastatic urothelial carcinoma receiving first-line platinum-based chemotherapy: retrospective international study of invasive/advanced cancer of the urothelium (RISC). Eur Urol. (2017) 71(2):281–9. 10.1016/j.eururo.2016.09.042 27726966 PMC5576985

[15] Yip PL Lee SF Choi CH Chan PS Cheung KA Chow CJ External validation of a nomogram to predict survival and benefit of concurrent chemoradiation for stage II nasopharyngeal carcinoma. Cancers (Basel). (2021) 13(17):4286. 10.3390/cancers13174286 34503096 PMC8428339

[16] Raghav K Hwang H Jácome AA Bhang E Willett A Huey RW Development and validation of a novel nomogram for individualized prediction of survival in cancer of unknown primary. Clin Cancer Res. (2021) 27(12):3414–21. 10.1158/1078-0432.CCR-20-4117 33858857 PMC8197749

[17] Chang YR Huang WK Wang SY Wu CE Chen JS Yeh CN. A nomogram predicting progression free survival in patients with gastrointestinal stromal tumor receiving sunitinib: incorporating pre-treatment and post-treatment parameters. Cancers (Basel). (2021) 13(11):2587. 10.3390/cancers13112587 34070456 PMC8197516

